# Anticholinergic exposure and its association with dementia/Alzheimer's disease and mortality in older adults

**DOI:** 10.1186/s12877-023-04095-7

**Published:** 2023-06-30

**Authors:** Insiya B. Poonawalla, Yihua Xu, Rainelle Gaddy, Alex James, Matt Ruble, Salina Burns, Suzanne W. Dixon, Brandon T. Suehs

**Affiliations:** 1 Humana Healthcare Research, Humana Inc., 500 W Main St, Louisville, KY 40202 USA; 2 Humana Pharmacy Solutions, Humana Inc., 500 W Main St, Louisville, KY 40202 USA

**Keywords:** Anticholinergic, Dementia, Alzheimer’s disease, Mortality, Beers criteria

## Abstract

**Background:**

Use of anticholinergic (ACH) medications is associated with increased risk of cognitive decline in the elderly. However, little is known about this association from a health plan perspective.

**Methods:**

This retrospective cohort study used the Humana Research Database to identify individuals with at least one ACH medication dispensed in 2015. Patients were followed until incidence of dementia/Alzheimer’s disease, death, disenrollment or end of December 2019. Multivariate Cox regression models were used to assess the association between ACH exposure and study outcomes, adjusting for demographics and clinical characteristics.

**Results:**

A total of 12,209 individuals with no prior ACH use or dementia/Alzheimer’s disease diagnosis were included. As ACH polypharmacy increased (i.e., from no ACH exposure, to one, two, three, and four or more ACH medications), there was a stair-step increase in the incidence rate of dementia/Alzheimer’s disease (15, 30, 46, 56 and 77 per 1,000 person-years of follow-up) and in the incidence of mortality (19, 37, 80, 115 and 159 per 1,000 person-years of follow-up). After adjusting for confounders, ACH exposure to one, two, three and four or more ACH medications was associated with a 1.6 (95% CI 1.4–1.9), 2.1 (95% CI 1.7–2.8), 2.6 (95% CI 1.5–4.4), and 2.6 (95% CI 1.1–6.3) times, respectively, increased risk of a dementia/Alzheimer’s disease diagnosis compared to periods of no ACH exposure. ACH exposure to one, two, three and four or more medications was associated with a 1.4 (95% CI 1.2–1.6), 2.6 (95% CI 2.1–3.3), 3.8 (95% CI 2.6–5.4), and 3.4 (95% CI 1.8–6.4) times, respectively, increased risk of mortality compared to periods of no ACH exposure.

**Conclusions:**

Reducing ACH exposure may potentially minimize long-term adverse effects in older adults. Results suggest populations which may benefit from targeted interventions to reduce ACH polypharmacy.

## Background

The 2015 American Geriatrics Society Beers Criteria recommend against concurrent use of three or more central nervous system and two or more anticholinergic (ACH) medications in the elderly due to increased risk of cognitive decline [1]. The updated 2019 Beers Criteria for potentially inappropriate medication use in older adults added drugs with strong ACH properties to their list based on more recent evidence [2], which continues to accumulate to support these recommendations. For example, in a diverse population of 6,249 Latino adults residing in four major U.S. cities, ACH drug use at two points in time during an average 7-year follow-up was associated with lower global cognition, learning, memory, and executive functioning [3]. Additionally, among 8,216 older adults in Great Britain, recurrent use of ACH medications that were scored 3 on the Anticholinergic Cognitive Burden (ACB3) scale was linked with dementia by year 10 of cohort follow-up [4]. However, the impact on dementia was not observed in patients on ACH medications characterized with a lower anticholinergic burden of ACB 1 or 2 or in those who had no prior use.

Due to the possible negative effects of ACH exposure, medications with higher ACH activity such as oxybutynin now include a caution for patients with preexisting dementia treated with cholinesterase inhibitors because adding ACH drugs may exacerbate cognitive symptoms [5]. Prospective data also support the connection between increasing ACH exposure and increasing rates of cognitive decline and dementia in older populations [6], and two recent systematic reviews and meta-analyses found the risk of incident dementia and cognitive decline increased with increasing ACH exposure [5, 7]. Despite these potential risks, older individuals may be initiated to or continued on these agents because the ACH medication class is broad and includes therapies for several conditions affecting older individuals such as depression [8], overactive bladder [9, 10], and Parkinson’s disease [11].

The issue of polypharmacy may further complicate this clinical picture when assessing the possible risks and benefits of ACH medications for older adults. Clinical consequences of polypharmacy in older adults include cognitive impairment, disability, falls, frailty, increased healthcare use, and mortality [12]. For example, an Italian multicenter cohort study found approximately half of the cohort of 342 older adults with mild cognitive impairment took three or more drugs per day, and at the end of one year, the odds of dementia were six times higher for those taking more than three medications compared with similar adults taking fewer than three drugs. Further evidence demonstrated dementia risk was five times higher for those assessed with Anticholinergic Risk Scale (ARS) ≥ 1 [13]. In another example, a polypharmacy study of a population of patients aged ≥ 65 years from an outpatient multi-morbidity clinic in Australia found exposure to polypharmacy in the sedative and ACH medication classes in particular was linked to future mortality [14].

While the impact of ACH burden on cognition is well documented globally [15–19], less is known regarding the prevalence and intensity of ACH use in large, U.S. health plan populations, and of how ACH polypharmacy in particular may affect the risk of dementia, Alzheimer’s disease, and mortality in this population. Health plans may play a unique role in helping to mitigate medication-related problems such as polypharmacy in the elderly, adding further value to considering the issue through a health plan lens. To address this issue, we used real-world claims to describe the demographics and clinical characteristics of a large population of Medicare Advantage prescription drug (MAPD) plan beneficiaries newly dispensed ACH medications and analyzed the relationship between polypharmacy and cumulative ACH therapy exposure on incident dementia/Alzheimer’s disease and mortality.

## Methods

### Study design

**Fig. 1:**
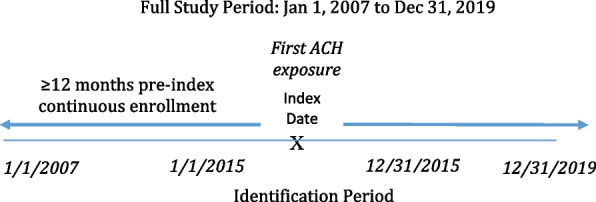
Study design

This retrospective cohort study used the Humana Research Database to identify MAPD patients, 65–89 years of age, newly initiated on an ACH medication with an Anticholinergic Cognitive Burden scale (ACB) score 2 or 3 [20, 21] between January 1, 2015 and December 31, 2015 (index date = first ACH exposure). The cut point of 89 years was chosen to reflect organizational data and privacy policies requiring exclusion of patients 90 years and older from research studies. The ACB score is a high-quality anticholinergic burden scale [22] and is based on a systematic review by Boustani et al. which ranks drugs from a scale of one to three [20]. An ACB score of 2 or 3 indicates drugs with established and clinically relevant cognitive ACH effects, as compared to a score of one, which indicates drugs with potential ACH effects. Eligible patients had 12-months pre-index continuous enrollment, and were followed up until the end of enrollment, death, or end of study period (December 31, 2019) whichever occurred first (Fig. 1).

Patients with any exposure to a prescription ACH medication or any evidence of a diagnosis or medication use for dementia/Alzheimer’s disease any time prior to 2015 (until 2007) were excluded from the study.

### Study exposure

Using pharmacy claims data, we measured time-varying ACH exposure to account for changes in medication regimens over extended periods of follow-up. ACH exposure was measured in two ways: i. Time-varying person-time level ACH polypharmacy measure for medication exposure on each follow-up day (i.e., no exposure, 1, 2, 3, ≥ 4 ACH medications), and ii. Cumulative ACH exposure measure derived by summing each person’s standardized daily ACH exposure intensity during the follow-up, and categorized as low, moderate, high and very high.

Exposure intensity was measured taking into account both drug-specific properties (i.e., anticholinergic activity) and patient-specific dosing. It was measured with medication coverage arrays using Defined Daily Dose (DDD) [23] to standardize dosing across different medications and the drug-specific ACB score to provide a scale by strength of anticholinergic activity. The DDD is the assumed average maintenance dose per day for a drug used for its main indication in adults. For each anticholinergic dispensing, the Standardized Daily Dose (SDD) of the medication was calculated by taking the estimated number of dosage units consumed in a day multiplied by the unit strength then divided by the DDD, as described by the following equation:

$$\mathrm{SDD}=\lbrack(\mathrm{Number}\;\mathrm{of}\;\mathrm{units}\;\mathrm{dispensed}/\mathrm{days}'\;\mathrm{supply})\ast\mathrm{unit}\;\mathrm{dose}\rbrack/\mathrm{DDD}$$

For example, if 90 tablets of oxybutynin-immediate release 5 mg were dispensed with a days’ supply of 30, where the DDD for oxybutynin is 15 mg, then the calculation would be: [(90 tablets/30 days’ supply)*5 mg]/15 mg = 1.0 SDD. A value of 1.0 SDD indicates that for the period of time covered by the prescription dispensing, the patient received 1.0 standardized dose per day covered with medication. Once the SDD for an individual dispensing is determined, the SDD was multiplied by the ACB scale score of the medication dispensed to yield a drug and patient–specific measure of the *Standardized Daily Anticholinergic Exposure* (SDACE)*.* This value was input into a medication days’ coverage array for the period of time including the index date through the last day covered based on the days’ supply for the dispensing. A grace period of 15 days’ supply was added to each anticholinergic dispensing to account for partial adherence to treatment and residual anticholinergic effect, unless the days’ supply is ≤ 7 then 3 days was added. Using array-based methods, the drug-specific SDACE was aggregated at the patient level to account for coverage with multiple medications on a given day, yielding a *Summated Standardized Daily Anticholinergic Exposure* (SumSDACE) measure that reflected the total anticholinergic intensity of exposure for each patient on each day of follow-up, taking into account all anticholinergic medication that the individual was receiving.

Cumulative anticholinergic exposure was then measured as the cumulative SumSDACE for all preceding days during the follow-up period. Breakpoints for categorization of cumulative anticholinergic exposure (i.e., into low, moderate, high, and very-high cumulative exposure categories) were determined using all post-index follow-up times and were based on initial assessment of the distribution of the data and then informed by clinical judgment and existing literature.

### Study outcomes

The dementia/Alzheimer’s disease study outcome was defined by the presence of ≥ 2 relevant diagnosis codes on medical claims or by the presence of ≥ 1 relevant diagnosis code on a medical claim and ≥ one prescription claim for a cognition-enhancing medication (rivastigmine, galantamine, donepezil or memantine). Mortality was measured using information from the enrollment data provided by the Centers for Medicare and Medicaid Services.

### Statistical analysis

Crude incidence rates were reported as rate per 1,000 person-years. Since patients dynamically start and stop treatment with ACH medications over the course of an extended period of follow-up, a series of extended Cox regression models were computed accounting for time-varying ACH exposure when examining the relationship to incident dementia/Alzheimer’s disease and mortality outcomes. Regression models were adjusted for patient demographics, Elixhauser Comorbidity Index [24–26], RxRisk-V score [27], baseline medication use, baseline healthcare resource use, and time-varying use of other high-risk non-ACH medications associated with cognitive impairment (i.e., non-ACB drugs included in the drug classes anxiolytic benzodiazepines, anticonvulsant benzodiazepines, anti-depressants, anti-convulsants, anti-psychotics, barbiturate hypnotics, sedative hypnotics, anti-Parkinson drugs and anti-nausea/vertigo agents). [28–31] The Elixhauser comorbidity index uses 31 categories of ICD-9 and ICD-10 diagnosis codes [24, 26] to calculate a score that is associated with hospital charges, length of stay, and mortality [26]. The RxRisk score is a pharmacy-based measure of comorbidity burden that was originally developed to assess medication burden in the Veterans Health Administration (VHA) population [27], ranges from 0–45, and has been shown to be predictive of healthcare costs and mortality in a range of populations [27, 31–38]. Two of the original RxRisk-V categories (neurogenic bladder and ostomy) are not included in the current implementation because products associated with these conditions (urinary catheters and colostomy supplies, respectively) are not captured in outpatient pharmacy claims. Hence the composite score in the present study ranges from 0 to 43.

## Results

**Fig. 2:**
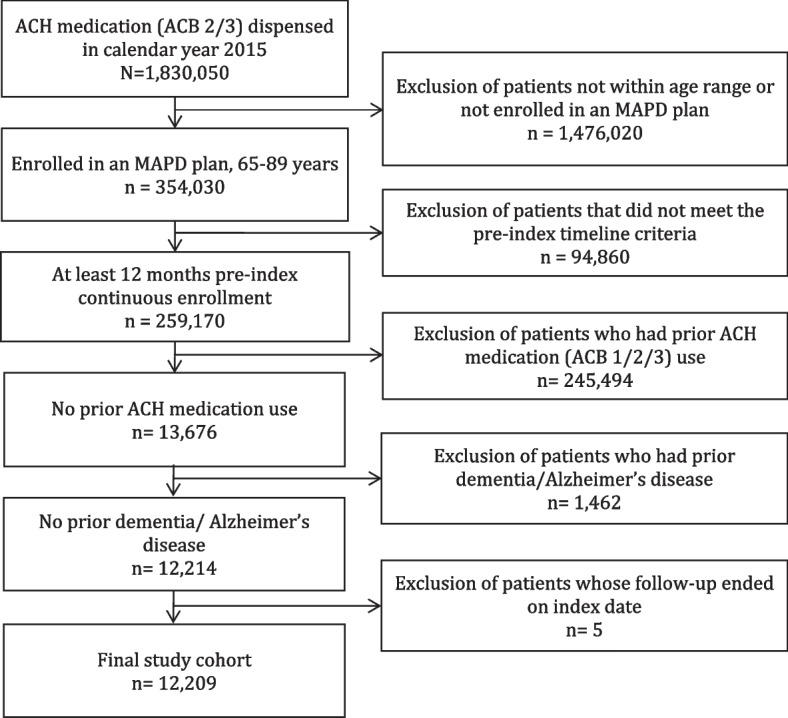
Attrition diagram

We identified 12,209 individuals with no prior ACH therapy use and no prior diagnoses of dementia or Alzheimer’s disease (Fig. 2). The cohort was 57.6% female, 80.6% White, 13.2% with low-income subsidy and dual Medicare-Medicaid eligibility status, with a mean age of 72.2 years. Prevalence of non-ACH high-risk medication use was 51.2% (Table 1). We observed maximum concurrent ACH use of one, two, three, or four or more therapies during the follow-up period in 59%, 30%, 8%, and 3% of the cohort, respectively. Individuals exposed to four or more ACH medications had the highest baseline utilization of inpatient stays (16.7%) and emergency department visits (29.7%, Table 1).

**Table 1:** Baseline demographic and clinical characteristics, by highest ACH polypharmacy exposure attained

		**Anticholinergic Polypharmacy**				
**Variables**	**Total**	**1 medication**	**2 medications**	**3 medications**	**4 + medications**	***P*** ** value**
N	12,209	7,153	3,708	1,001	347	
Age, mean [SD]	72.2 [5.6]	72.3 [5.5]	72.0 [5.7]	72.3 [5.8]	72.4 [6.1]	0.0049
Gender, n(%)						
Female	7,038 (57.6)	4,089 (57.2)	2,138 (57.7)	602 (60.1)	209 (60.2)	0.2429
Male	5,168 (42.3)	3,063 (42.8)	1,569 (42.3)	398 (39.8)	138 (39.8)	
Race/Ethnicity, n(%)						
White	9,843 (80.6)	5,712 (79.9)	3,007 (81.1)	826 (82.5)	298 (85.9)	0.0182
Black	1,497 (12.3)	927 (13.0)	424 (11.4)	112 (11.2)	34 (9.8)	
Other/unknown	869 ( 7.1)	514 (7.2)	277 (7.5)	63 (6.3)	15 (4.3)	
Geographic Region, n(%)						
Northeast	295 (2.4)	184 (2.6)	77 (2.1)	26 (2.6)	< 10	0.0177
Midwest	2,192 (18.0)	1,245 (17.4)	694 (18.7)	176 (17.6)	77 (22.2)	
South	8,150 (66.8)	4,792 (67.0)	2,443 (65.9)	677 (67.7)	238 (68.6)	
West	1,569 (12.9)	931 (13.0)	493 (13.3)	121 (12.1)	24 (6.9)	
Low Income/ Dual Eligibility Status, n(%)						
Low income subsidy (LIS) Only	565 (4.6)	323 (4.5)	163 (4.4)	51 (5.1)	28 (8.1)	< 0.001
Dual eligibility (DE) Only	< 10	< 10	< 10	< 10	0 (0.0)	
LIS and DE	1,608 (13.2)	874 (12.2)	482 (13.0)	170 (17.0)	82 (23.6)	
Elixhauser comorbidity index, mean [SD]	1.7 [1.8]	1.6 [1.7]	1.8 [1.8]	2.0 [2.0]	2.5 [2.5]	< 0.001
Rx Risk-V score, mean [SD]	3.4 [2.2]	3.3 [2.1]	3.6 [2.2]	3.7 [2.4]	3.7 [2.7]	< 0.001
Non-ACH high-risk medication use^a^, n(%)	6,249 (51.2)	3,547 (49.6)	1,981 (53.4)	545 (54.4)	176 (50.7)	< 0.001
Pre-index inpatient visit n (%)	1,282 (10.5)	705 (9.9)	381 (10.3)	138 (13.8)	58 (16.7)	< 0.001
Pre-index emergency department visit n(%)	2,901 (23.8)	1,631 (22.8)	903 (24.4)	264 (26.4)	103 (29.7)	0.002

*ACH* Anticholinergic, *SD* Standard deviation

^a^Non-ACH high-risk medications included anxiolytic benzodiazepines, anticonvulsant benzodiazepines, antidepressants, anti-convulsants, anti-psychotics, barbiturate hypnotics, sedative hypnotics, anti-Parkinson drugs and anti-nausea/vertigo agents not included on the ACB scale

As ACH polypharmacy increased, there was a stair-step increase in the incidence of dementia/Alzheimer’s disease (Table 2) and in the incidence of mortality (Table 3). For example, moving from periods of no ACH exposure to 1, 2, 3 and ≥ 4 ACH medications, the incidence rate of dementia/Alzheimer’s disease increased from 15 per 1,000 person-years to 31, 46, 56 and 77 (Table 2) and the incidence rate for mortality increased from 19 per 1,000 person-years to 37, 79, 115 and 159 (Table 3), respectively. A similar stair-step increase in the incidence rate of both outcomes was observed as cumulative ACH exposure increased from moderate to high to very high compared to the period of low ACH exposure.

**Table 2:** Incidence rates and hazard ratios for the incidence of Dementia/Alzheimer's disease

Measure	Events	Person-time (years)	Crude IR (per 1,000 PY)	Adjusted Hazard Ratio (95% CI)
Overall	819	41,437	19.77	
**Anticholinergic polypharmacy**				
No ACH exposure	474	31,249	15.17	Reference
1 ACH meds	253	8290	30.52	1.64 (1.39—1.93)
2 ACH meds	69	1515	45.54	2.17 (1.67—2.83)
3 ACH meds	17	305	55.74	2.59 (1.54—4.36)
≥ 4 ACH meds	6	78	76.92	2.59 (1.06—6.32)
**Cumulative Anticholinergic Exposure**				
Low	386	22,246	17.35	Reference
Moderate	202	10,688	18.90	1.05 (0.88—1.25)
High	108	4424	24.41	1.17 (0.93—1.48)
Very High	123	4079	30.15	1.32 (1.04—1.68)

*IR* Incidence rate, *PY* Person years

**Table 3:** Incidence rates and hazard ratios for mortality

Measure	Events	Person-time (years)	Crude IR (per 1,000 PY)	Adjusted Hazard Ratio (95% CI)
Overall	1129	42,887	26.33	
**Anticholinergic polypharmacy**				
No ACH exposure	623	32,133	19.39	Reference
1 ACH meds	321	8680	36.98	1.42 (1.2—1.6)
2 ACH meds	131	1638	79.98	2.64 (2.1—3.3)
3 ACH meds	40	347	115.27	3.75 (2.6—5.4)
≥ 4 ACH meds	14	88	159.09	3.36 (1.8—6.4)
**Cumulative Anticholinergic Exposure**				
Low	494	22,752	21.70	Reference
Moderate	279	11,051	25.20	1.21 (1.01—1.41)
High	162	4655	34.80	1.51 (1.25—1.83)
Very High	194	4429	43.80	2.11 (1.76—2.52)

*IR *Incidence rate, *PY *Person years

After adjusting for confounders, ACH exposure to one, two, three and four or more ACH medications was associated with a 1.6 (95% CI 1.4–1.9), 2.1 (95% CI 1.7–2.8), 2.6 (95% CI 1.5–4.4), and 2.6 (95% CI 1.1–6.3) times, respectively, increased risk of a dementia/Alzheimer’s disease diagnosis compared to periods of no ACH exposure (Table 2). Similarly, ACH exposure to one, two, three and four or more medications was associated with a 1.4 (95% CI 1.2–1.6), 2.6 (95% CI 2.1–3.3), 3.8 (95% CI 2.6–5.4), and 3.4 (95% CI 1.8–6.4) times, respectively, increased risk of mortality compared to periods of no ACH exposure (Table 3).

## Discussion

Greater ACH burden was associated with an increased rate of dementia/Alzheimer’s disease and mortality in this study of seniors enrolled in a Medicare Advantage health plan. While the magnitude of effect differed across our results, the directionality is in line with a recent meta-analysis that documented a 46% increased odds of incident dementia in patients with ≥ 3 months of ACH medication use [5]. Additionally, reflecting on our results, a Cochrane Database review and meta-analysis with 968,428 participants in total suggested a possible doubling of the risk of dementia with ACH drug exposure and that a greater ACB score increases the risk of dementia [39]. Furthermore, two other systematic reviews and meta-analyses reported a positive association between ACH burden and risk of mortality across the majority of the included studies spanning from 1990 to 2018 [40, 41]. The authors of those studies acknowledged short follow-up time as an important limitation, with only a few of the studies meeting the criteria for superior quality. In our study, we were able to follow patients for four years enabling a more robust data collection in comparison to many existing studies. With this longer-term evaluation, we found the risk of cognitive decline and mortality with ACH exposure may apply across a wide range of populations and settings.

Additionally, we considered the intersection of polypharmacy with ACH drug exposure and found that significant portions of this population were prescribed more than one ACH medication. Of note, 30% of this population was prescribed two or more ACH therapies, 8% used three or more, and 3% used four or more drugs with ACH activity on at least one day during their follow-up. These results should be concerning, as a recently published meta-analysis of 2.9 million elderly individuals reported a 28% increased risk of mortality associated with use of five or more drugs [42]. These results represent a significant number of older individuals exposed to the well-documented risks of cognitive decline and increased mortality that are associated with this therapeutic class of medications [43–45]. Health plans have an opportunity and important role in minimizing ACH exposure for enrolled patients. For example, coordinated educational outreach efforts can be made to pharmacy, provider, and patients on the safety concerns with the use of multiple anticholinergics.

While we did not specifically assess the impact of ACH therapy on other treatment outcomes, more than 50% of our cohort was exposed to non-ACH, high-risk therapy. These results point toward the importance of assessing both ACH and total polypharmacy in future research and when designing de-prescribing programs with the intention of lowering the risk of cognitive decline. Future research should continue to further define these relationships and provide insights into optimal policies and programs to address ACH exposure/polypharmacy in the elderly, particularly with the understanding that cognitive decline and its attendant sequelae can lead to a loss of independence [46].

### Limitations

Dementia may not be well represented in administrative claims data, and diagnosis codes do not provide information on severity of dementia. This study used a broad range of diagnosis codes and an extended follow-up period (3–5 years) to identify new onset cases. Additionally, this study focuses only on prescription medication utilization which is well captured using claims data; however, over-the-counter medications, such as some antihistamines, may have strong anticholinergic effects and are not captured in prescription claims, which would lead to underestimating ACH polypharmacy. It is not possible to know from claims data if the drug was indicated for as needed versus daily use. Hence, DDD was an assumed average maintenance dose per day for a drug used for its main indication in adults. We assumed that a patient took the medications based on the days’ supply variable. This may lead to an overestimate of ACH exposure. Similar to other retrospective database studies, this study was subject to limitations including coding errors of omission and commission, incomplete claims, unreliable clinical coding, and unobservable factors that also may have influenced the outcomes. For example, while pharmacy claims and days’ supply on prescription fills indicate the possession of pharmacotherapy, we are unable to verify if the patient took the medication. This study used data from a single data source, and as such the results may not be generalized to the general population. However, Humana is a large national health plan with beneficiaries residing in a broad array of geographic regions.

## Conclusions

ACH medication use is common among health plan members residing in broad geographic regions of the United States. ACH drugs were associated with significantly increased risk of dementia and mortality in this real-world population of older adults. Reducing ACH exposure may potentially minimize long-term adverse effects in older adults. Results suggest populations which may benefit from targeted interventions to reduce ACH polypharmacy.

## Data Availability

The datasets generated and analyzed during the current study are not publicly available due the proprietary nature of the work but may be available in summary form from the corresponding author on reasonable request.

## Abbreviations

ACB: Anticholinergic Cognitive Burden
ACH: Anticholinergic
ARS: Anticholinergic Risk Scale
COPD: Chronic obstructive pulmonary disease
DDD: Defined Daily Dose
SDACE: Standardized Daily Anticholinergic Exposure
SDD: Standardized Daily Dose
SumSDACE: Summated Standardized Daily Anticholinergic Exposure
